# Manganese-enhanced MRI in the evaluation of cell-based therapy for myocardial restoration

**DOI:** 10.1186/1532-429X-14-S1-P50

**Published:** 2012-02-01

**Authors:** Paul J Kim, Ildiko Toma, INing E Wang, Phillip C Yang

**Affiliations:** 1 Cardiovascular Medicine, Stanford, Stanford, CA, USA

## Background

To date, the underlying mechanism responsible for the restoration of the injured myocardium following transplantation of stem cells has not been clearly identified. Three major hypotheses have been previously proposed: cardiac differentiation of transplanted cells (de novo myocardial regeneration), paracrine effect on existing myocardium (myocardial salvage) or recruitment of cardiac progenitor cells. Manganese-enhanced MRI (MEMRI) has recently been published as a reliable method of imaging viable myocardium (Dash et al 2011). Utilizing MEMRI, we evaluated the changes in the viability of the injured myocardium to further investigate the underlying mechanism of functional restoration using stem cell therapy.

## Methods

Thirteen Fox Chase SCID Beige mice were subjected to permanent left anterior descending (LAD) ligation to create the mouse myocardial infarction model. 2.5 x 10^5 reporter-gene transduced embryonic stem cells (ESC-RGs) containing firefly luciferase (fluc) were subsequently transplanted into the intra-infarct region in eleven mice. Two mice were injected with normal saline into the intra-infarct region to serve as controls. 3T cardiac MRI was performed weekly for 4 weeks following LAD ligation and ESC-RGs transplantation to obtain LVEF measurements and MEMRI images. Additionally, bioluminescence images (BLI) were obtained weekly utilizing the transduced fluc gene to demonstrate persistent viability of the ESC-RGs. At selected time points (weeks 2, 3 and 4), the hearts were explanted, sectioned along the short axis plane and processed for H&E staining. The H&E stained slides provided histological correlation of MEMRI and bioluminescence images.

## Results

**Figure 1:**
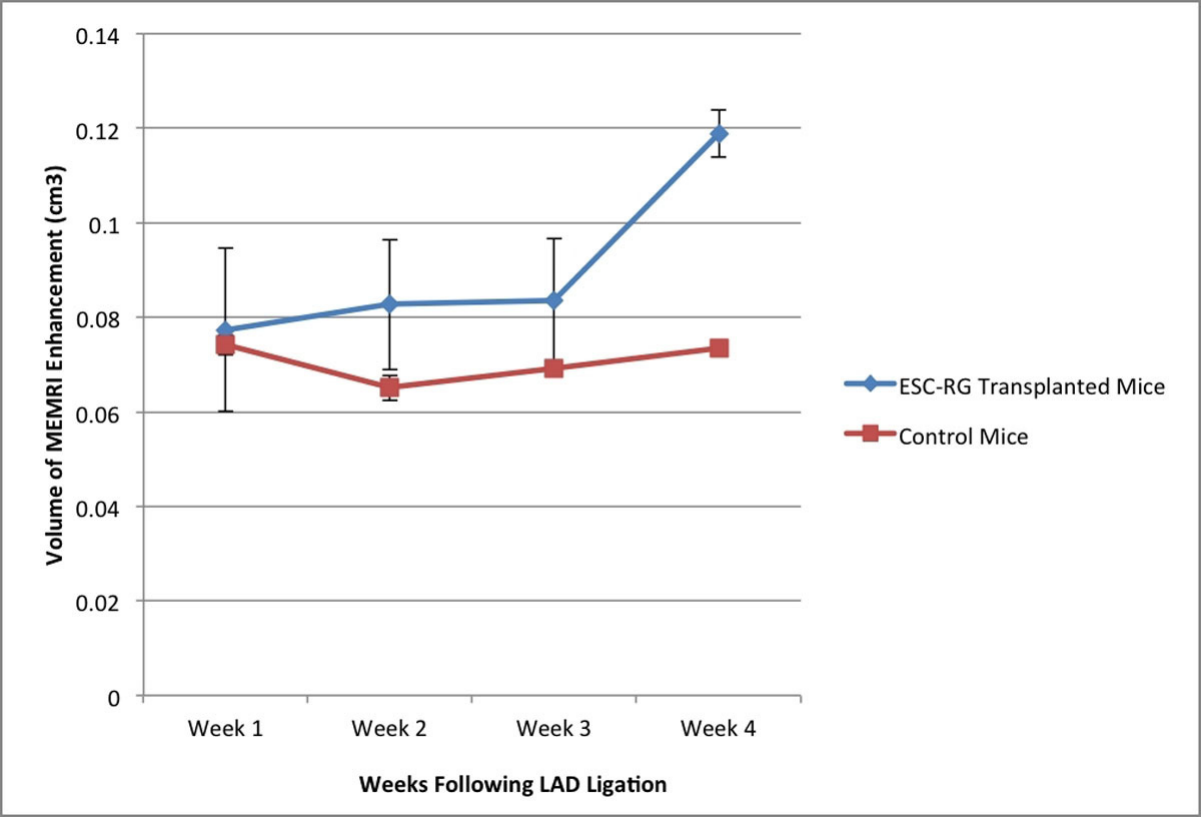
MEMRI volumes in ESC-RG transplanted vs control mice.

**Figure 2:**
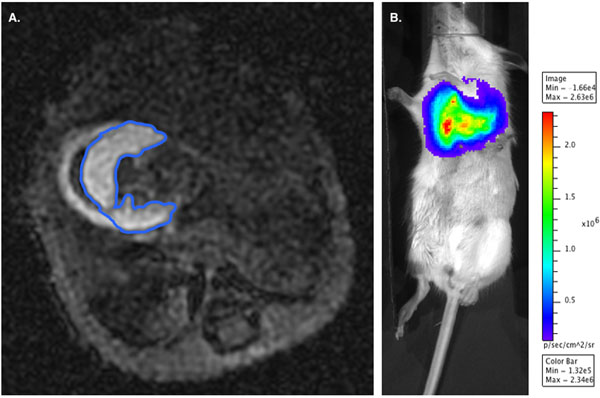
A) MEMRI enhancement of viable myocardium in an ESC-RG transplanted mouse B) BLI of transplanted viable ESC-RGs.

We demonstrate a trend towards improved LVEF with ESC-RGs transplanted hearts, consistent with the results of our group’s previously published data. The control group, in contrast, demonstrates no functional improvement with a stable but depressed LVEF following LAD ligation. A more sensitive measurement of myocardial restoration is significantly increased MEMRI signal observed in the ESC-RGs vs. control mice (.119+.005 cm3 vs .0736+.001 cm3 respectively, p=0.034), indicating improved myocardial viability (Figure 1). BLI confirmed the presence as well as engraftment of the transplanted ESC-RGs, which were confirmed histologically (Figure 2).

## Conclusions

This study demonstrates the functional improvement in ESC-RG transplanted mice. In addition, MEMRI shows a significant increase in viable myocardium in ESC-RG transplanted hearts, indicating myocardial restoration. This finding may support the hypothesis that functional improvement with stem cell therapy may be due to myocardial salvage.

## Funding

T32 CVIS Grant under National Institute of Biomedical Imaging and Bioengineering.

